# Mini-open technique for sternoclavicular joint resection for the treatment of osteoarthritis–a technical note

**DOI:** 10.3389/fspor.2026.1716440

**Published:** 2026-08-05

**Authors:** Maximilian Hinz, Agahan Hayta, Alp Paksoy, Rony-Orijit Dey Hazra, David A. Back, Doruk Akgün

**Affiliations:** Center for Musculoskeletal Surgery, Charité – Universitätsmedizin Berlin, Corporate Member of Freie Universität Berlin and Humboldt-Universität zu Berlin, Berlin, Germany

**Keywords:** instability, osteoarthritis, resection, shoulder, sternoclavicular

## Abstract

Sternoclavicular joint (SCJ) osteoarthritis may lead to debilitating pain and decreased activity, particularly during overhead motion. In case of failed nonoperative treatment, surgical management may be indicated. Good to excellent outcomes have been reported following SCJ resection. There generally exists, however, a high threshold for performing SCJ resection due to the rarity of this condition, the proximity to the mediastinal structures as well as concerns for postoperative instability. In this technical note, a mini-open technique for SCJ resection that is safe, reproducible and reliable is presented and discussed.

## Introduction

Osteoarthritis (OA) of the sternoclavicular joint (SCJ) is a common incidental finding in the aging patient (1). Symptomatic SCJ OA is predominantly found in middle-aged women and may lead to pain, tenderness, swelling, redness and limitations in range of motion (2–4). In cases that do not resolve with non-steroidal anti-inflammatories and physical therapy, medial clavicle excision may indicated (3, 5, 6). One of the earliest techniques for open medial clavicle excision was reported by Bremner more than 60 years ago (4). Bremner reported good outcomes in a small case series following resection of the medial inch of the clavicle (4). This technique has evolved since through a less aggressive resection (7–12) and even arthroscopic approaches (13–15). Regarding the extent of the resection, specifically, a parallel resection of 5 mm of bone from the medial clavicle, has been shown to achieve an adequate joint decompression while preserving the costoclavicular ligament as to avoid postoperative SCJ instability (7–13). An anatomical study, however, showed that only the inferior two-thirds of the medial end of the clavicle are covered by articular cartilage (7), suggesting that resection of this portion alone may provide adequate decompression (14). Supporting this concept, a clinical study evaluating the outcomes following arthroscopic excision of the intra-articular lower two-thirds of the medial end of the clavicle with the adjacent SCJ disc reported excellent outcomes, again indicating that limited resection may be sufficient for decompression (14). Nonetheless, this arthroscopic approach has not been widely adopted. In addition to the learning curve associated with arthroscopy for this relatively rare pathology, there is generally a high threshold for surgical intervention at the SCJ (14). Surgeons may also hesitate because of the proximity of the joint to the mediastinal structures (16). In this article, an easily reproducible, mini-open technique for SCJ resection is presented that utilizes a resection of the lower two-thirds of the medial end of the clavicle while preserving the posterior capsule of the SCJ as well as the costoclavicular ligament to minimize the risk for postoperative instability.

## Surgical technique

**Figure 1:**
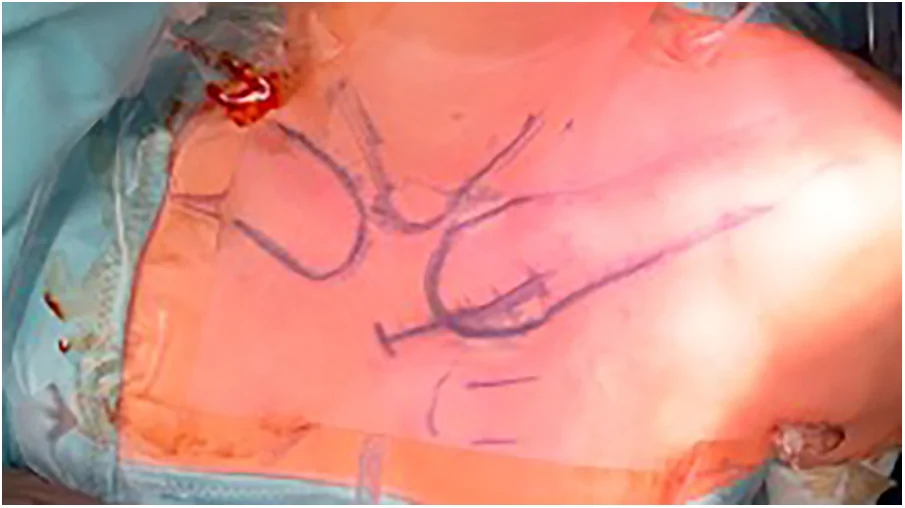
Anatomic landmarks, including both clavicles, the jugular notch and the medial borders of the SCM tendons, are marked. A 5 cm incision is made along the inferior third of the distal clavicle and the SCJ. SCJ, sternoclavicular joint; SCM, sternocleidomastoid.

**Figure 2:**
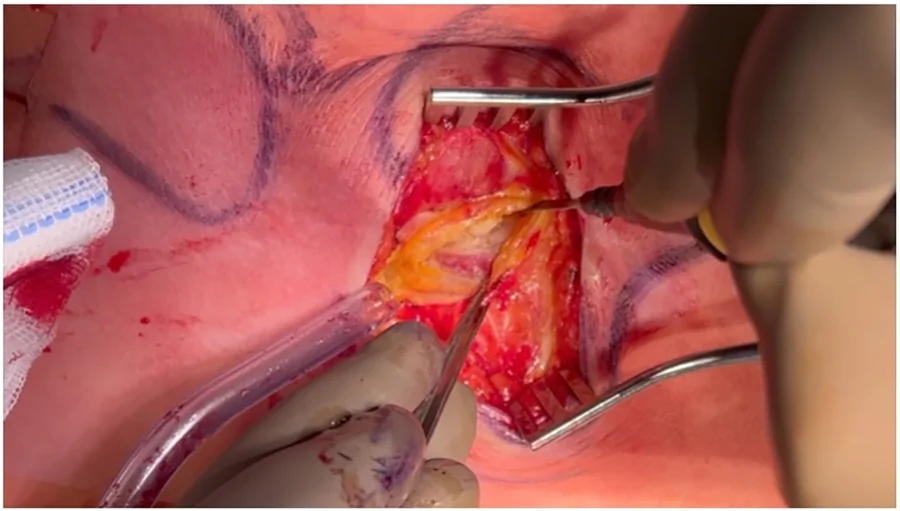
The SCJ capsule is split longitudinally using electrocautery. SCJ, sternoclavicular joint.

**Figure 3:**
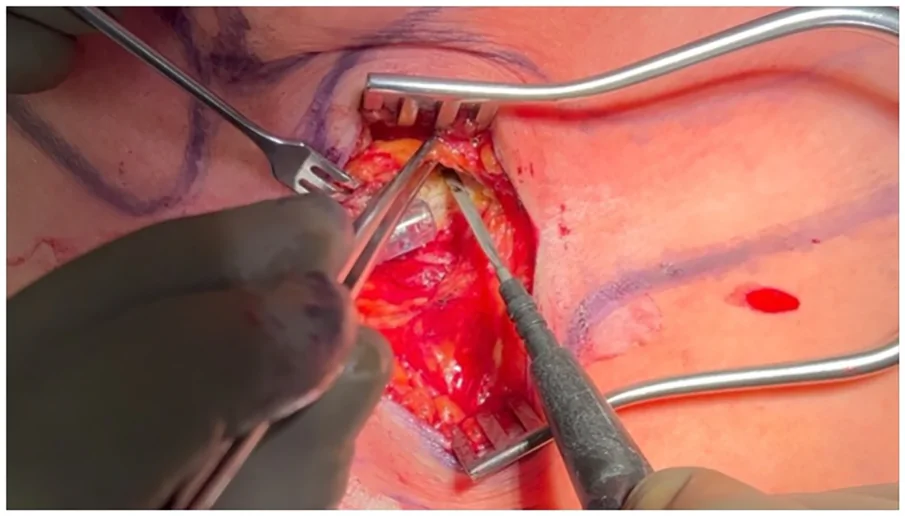
A subperiosteal dissection of the medial clavicle is performed.

**Figure 4:**
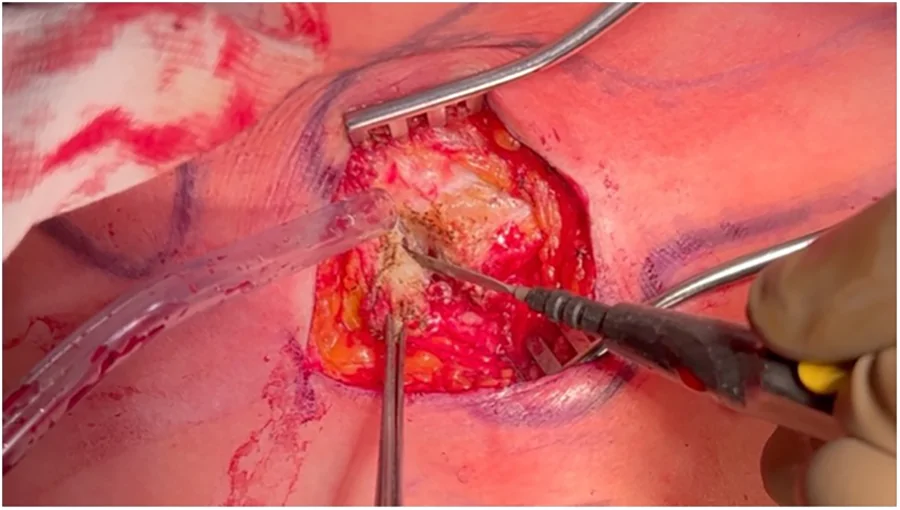
The degenerated SCJ disc is excised. SCJ, sternoclavicular joint.

**Figure 5:**
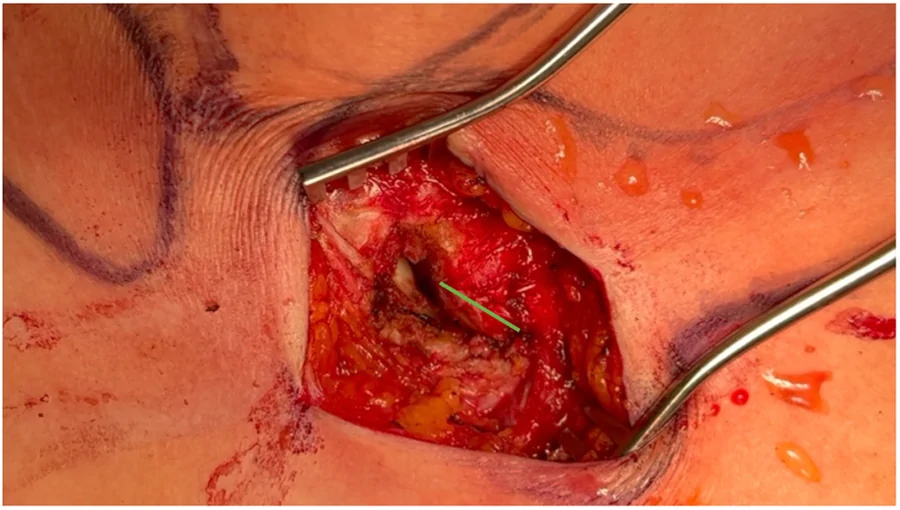
The level of resection level, the inferior two-thirds of the medial clavicle, is marked using electrocautery (green line).

**Figure 6:**
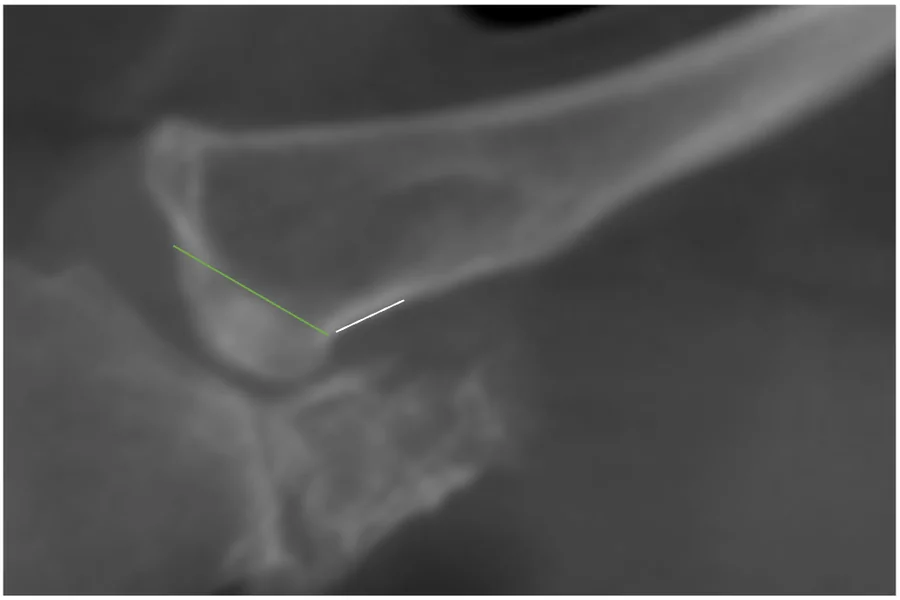
Preoperative dual-energy computed tomography of a left SCJ. The resection includes the inferior two-thirds of the medial clavicle (green line). During this, care is taken to preserve the costoclavicular ligament, which inserts approximately 10 mm lateral to the inferior sternoclavicular articular margin on the inferior aspect of the clavicle. SCJ, sternoclavicular joint.

**Figure 7:**
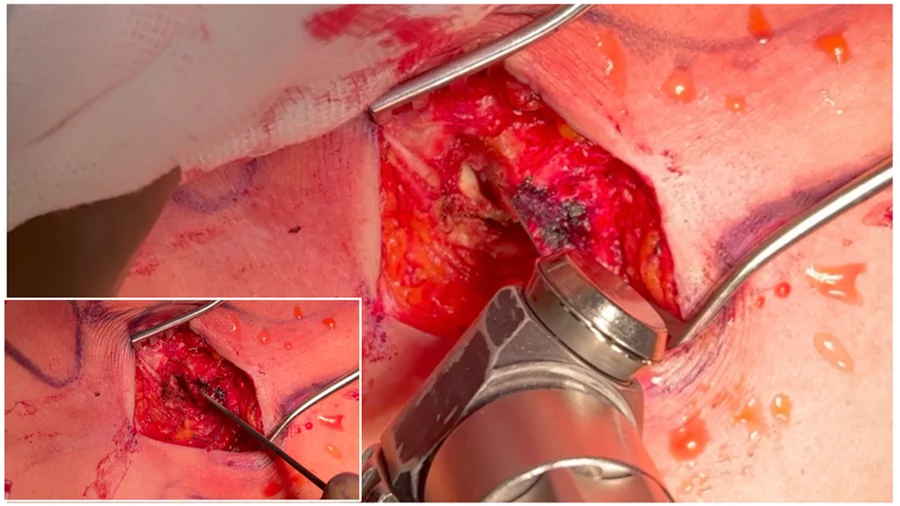
The anterior portion of the resection is performed using a saw and is completed using a chisel to protect the posterior SCJ capsule as well as the mediastinal structures (bottom left picture). SCJ, sternoclavicular joint.

**Figure 8:**
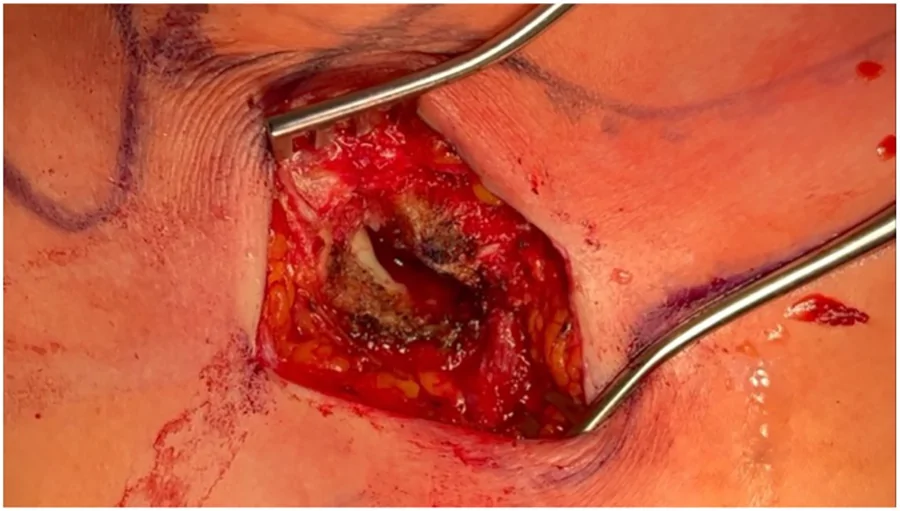
The SCJ has been adequately decompressed. SCJ, sternoclavicular joint.

After general anesthesia is induced, the patient is placed in the reverse Trendelenburg position with the head slightly tilted to the non-operative side. Anatomic landmarks, including both clavicles, the jugular notch, the medial borders of the sternocleidomastoid (SCM) tendons, as well as the incision, which is approximately 5 cm in length extending from the inferior third of the medial end of to the clavicle to the SCJ, are marked (Figure 1). Following the skin incision, examination under anaesthesia is performed with a clamp grasping the medial end of the clavicle to confirm that OA is the primary issue and instability is not present (5). During exposure, the overlying soft tissues as well as the SCM tendon are carefully protected (7). The SCJ capsule is then split longitudinally using electrocautery (Figure 2) and a subperiosteal dissection of the medial clavicle is performed (Figure 3). During this step, care is taken to preserve the costoclavicular ligaments and therefore, maintain stability (7, 10). Next, the degenerated SCJ disc is excised (Figure 4). The level of resection, which includes the lower two-thirds of the medial clavicle, is marked using electrocautery (Figure 5). The resection does not extend further than 10 mm laterally as to preserve the costoclavicular ligament (Figure 6) (7, 17). The anterior portion of the resection is performed using a saw. The resection is completed using a chisel to protect the posterior SCJ capsule, which is the most important restraint to anterior and posterior translation (18), as well as the mediastinal structures (Figure 7). Additionally, an often found inferior osteophyte on the medial clavicle may be removed using a rongeur. Following that, the SCJ is adequately decompressed (Figure 8). This is then also evaluated in full arm abduction. The surgical site is thoroughly irrigated and the SCJ capsule is closed in a pants-over-vest fashion to improve stability. The wound is closed in a layered fashion. Pearls and pitfalls of this technique are outlined in Table 1.

**Table 1:** Pearls of pitfalls of this technique.

**Step**	**Pitfalls**	**Pearls**
During surgical approach	Postoperative instability	Perform examination under anaesthesia using a clamp grasping the medial end of the clavicle to rule out instability.
Resection	Postoperative instability, injury to the mediastinal structures	Begin the resection using the saw, but complete it using a chisel as to protect the posterior SCJ capsule and mediastinal structures.
Resection	Postoperative instability	Do not resect the medial clavicle further than 10 mm laterally as to avoid compromise of the costoclavicular ligament.

SCJ, sternoclavicular joint.

## Postoperative rehabilitation

A shoulder sling is used for the first 2 postoperative weeks for comfort. During the first postoperative month, scapular protraction and retraction as well as cross-body arm adduction should be avoided. After the twelve-week postoperative follow-up, during which shoulder and scapulothoracic motion as well as pain are assessed, patients may be cleared to return to full activity.

## Discussion

This mini-open technique for SCJ resection enables an adequate resection of the articular part of the medial end of the clavicle while preserving the costoclavicular ligament, minimizing the risk of compromise of the retrosternal structures, and alleviating the need to learn SCJ arthroscopy, which may be associated with a shallow learning curve due to the rarity of this condition in itself as well as lack of other indications that SCJ arthroscopy may be used for.

In a recent systematic review, several different techniques have been reported for the treatment of SCJ OA with good to excellent outcomes reported between studies (19). In all but one study, 0.5 to 4 cm of bone of the medial clavicle were resected. An interposition of the sternal head of the SCM muscle into the joint space was added to the procedure in one study as a measure to prevent bony regrowth (20). In the remaining study, osteophyte resection alone without resection of the medial end of the clavicle was performed (21). The reported outcomes were overall good to excellent between studies and complications, particularly symptom persistence or recurrence as well as postoperative instability, were rare (5, 6, 19). Further, injury to the mediastinal structures was not reported by any study (19). Due to a lack of comparative studies, however, it is yet unclear which surgical technique would be associated with the most favorable outcomes. Techniques that involve a resection beyond 1 cm of the medial clavicle may, however, lead to postoperative instability (10).

Historically, resection of the medial end of the clavicle has also been performed for the treatment of SCJ instability, but was associated with poor functional outcomes (10, 12) and has been abandoned since. In case of instability, SCJ reconstruction or repair should be performed instead (22) with good outcomes reported at long-term follow-up (23).
